# Non-Contaminating Camouflage: Multifunctional Skin Microornamentation in the West African Gaboon Viper (*Bitis rhinoceros*)

**DOI:** 10.1371/journal.pone.0091087

**Published:** 2014-03-05

**Authors:** Marlene Spinner, Stanislav N. Gorb, Alexander Balmert, Horst Bleckmann, Guido Westhoff

**Affiliations:** 1 Functional Morphology and Biomechanics, Zoological Institute, Kiel University, Kiel, Germany; 2 Institute of Zoology, University of Bonn, Bonn, Germany; University of Akron, United States of America

## Abstract

The West African Gaboon viper (*Bitis rhinoceros*) has an extraordinary coloration of pale brown and velvety black markings. The velvety black appearance is caused by a unique hierarchical surface structures which was not found on the pale brown scales. In the present study we examined the wettability of the vipeŕs scales by measuring contact angles of water droplets. Velvet black scale surfaces had high static contact angles beyond 160° and low roll-off angles below 20° indicating an outstanding superhydrophobicity. Our calculations showed that the Cassie-Baxter model describes well wettability effects for these surfaces. Self-cleaning capabilities were determined by contaminating the scales with particles and fogging them until droplets formed. Black scales were clean after fogging, while pale scales stayed contaminated. Black scales feature multifunctional structures providing not only water-repellent but also self-cleaning properties. The pattern of nanoridges can be used as a model for surface-active technical surfaces.

## Introduction

**Figure 1:**
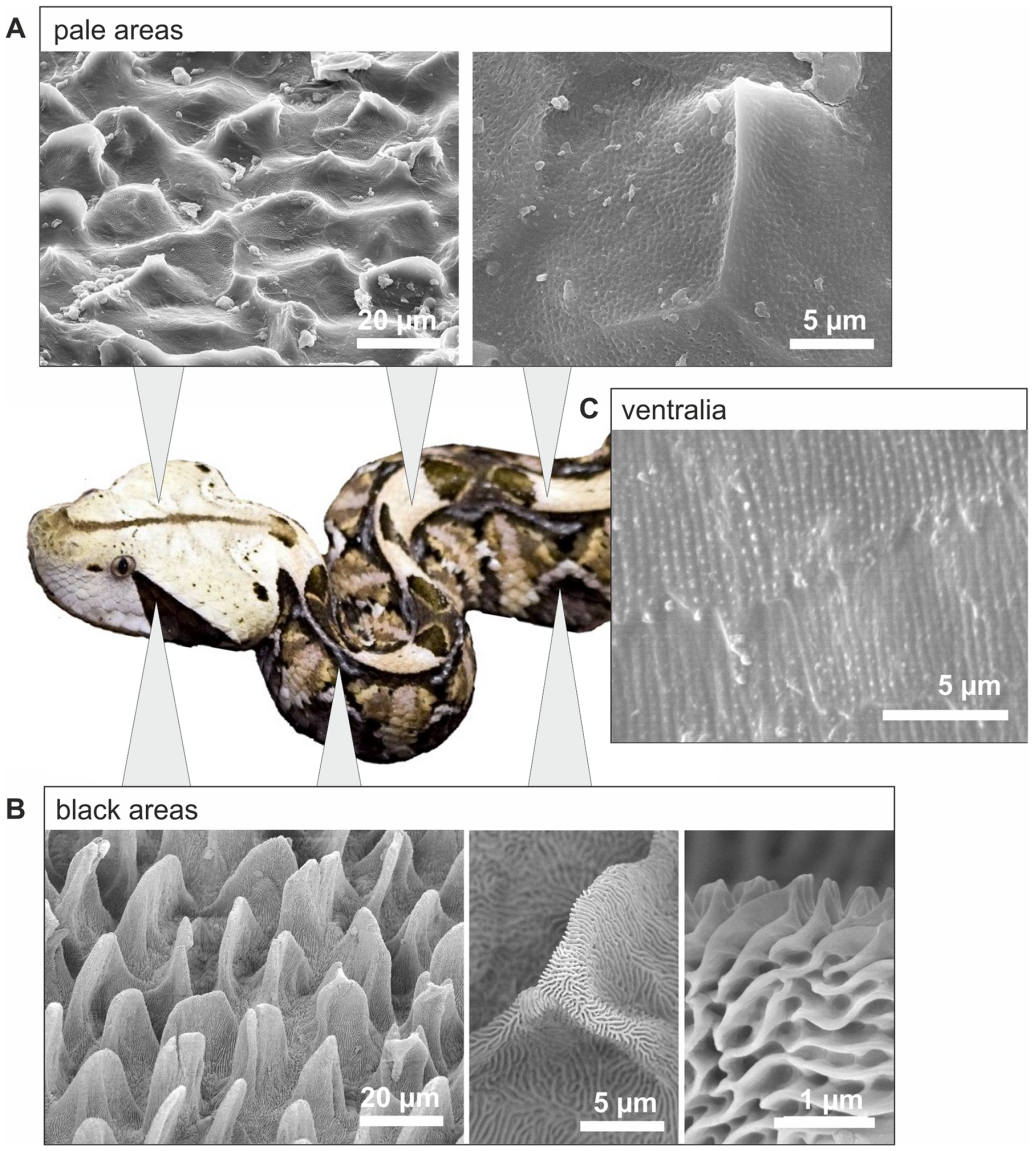
SEM-micrographs of the microornamentation (MO) of *Bitis rhinoceros* on different body regions. The MO patterns coincide with the black and pale colouration. **A,** Pale dorsal areas show a verrucate MO pattern covered with pits in nanometer range. **B,** Black dorsal areas show a hierarchical microornamentation consisting of microscopic leaf-like structures covered with nanoridges. **c,** Ventralia have nanoridges with nanoscopic knobs (after Spinner et al., 2013).

In our recent article we revealed a mechanism for black colour enhancement by epidermal microstructures in snakes [1]. Dorsal scales of the West African Gaboon viper, *Bitis rhinoceros*, feature two different patterns of structures (microornamentation, MO) that spatially coincide with the striking colouration of the snake. The geometric pattern consists of contrasting black and pale markings. Black scale areas have a unique hierarchically structured surface of microscaled leaf-like elevations and nanoridges, whereas pale scale areas were only slightly micro- and nanostructured (Fig. 1). Comparative optical measurements on black and pale scale sites revealed the contribution of these structures to viewing-angle independent minimization of reflectivity and maximization of light absorbance on the black areas. It has been shown that the reflecting and absorbing properties result from a combination of hierarchical structures impeding reflectance and dark pigments that absorb light. The high contrasting pattern of reflecting pale areas and light absorbing, low reflecting black areas, provides an astonishing camouflage for the large snakes on the ground of the African rainforest, where light and shade form a similar contrasting pattern. However, if we compare the snake scale geometry on velvet black scale surfaces with structures described in studies assessing wettability and self-cleaning properties, we can assume that the structure of these black areas might reveal water-repellent and self-cleaning properties, previously described in other biological and technical systems [2]–[10].

In this study we compared the wettability and self-cleaning properties between the velvet-black-coloured and the pale skin regions of *Bitis rhinoceros*. We determined the static contact angle of water droplets on both skin regions and calculated the surface free energy of the skin material on the nearly unstructured ventral scales without microstructures (Fig. 1). In calculation, the Cassie-Baxter wetting hypothesis was tested for black scales. For this purpose, we determined the solid area fraction of the surface structures of black scales and measured contact angles on ventral scales, having a relatively smooth surface, as a reference for the intrinsic contact angle of the snake skin. To determine the quality of self-cleaning properties, we contaminated dorsal skin areas with fluorescent micro-particles and subsequently fogged the skin with water. Finally, we quantified and compared the cleaning effect of pale skin parts and black skin parts.

## Material and Methods

### Animals

Contact angle measurements and self-cleaning experiments were performed on ventral and dorsal parts of an exuvium of an single individual of *Bitis rhinoceros* that was kept by a private owner. To confirm the results on a live specimen, the wetting properties were also investigated on an individual of *B. rhinoceros* that was kept at the Tierpark Hagenbeck (Tierpark Hagenbeck, gGmbH, Hamburg, Germany). The owners of the two snakes gave us the permission to use the shed skin of their animals for our study. Tierpark Hagenbeck allowed us to take pictures of their individual of *B. rhinoceros*.

### Contact angle measurements

To determine the wettability and surface free energy of the scales of *B. rhinoceros*, we measured contact angles of droplets of water, diiodomethane, and ethylene glycol on different scale types of the snake. Planar rectangular pieces of about 25 mm^2^ were cut out of the ventral and out of the black or pale dorsal scales of the exuvium. In accordance to Marmur [11] we used droplets that were two orders of magnitude higher than the microstructures. The upper limit of 1 µl for the drop volume was given by scale shape. The snake scales had keels and planar surfaces (of area of about 25 mm^2^) were only found on both sides of this structure. The droplets were placed on the scale pieces with the dispersion needle of the contact angle measuring system (OCA 20, Data Physics Instruments GmbH, Filderstadt, Germany). Measurements were conducted at indoor conditions (T = 20–24°C, relative humidity  = 45–55%). The scales were neither washed nor otherwise treated before the measurements. Contact angles on ten pieces of each of the respective scale types (ventral, dorsal black, dorsal) were measured. The needle in droplet method was used, when the droplets rolled off immediately after being placed on the respective surface. This was the case for the superhydrophobic surfaces (see discussion).

### Scanning electron microscopy and measurements of the solid area fraction

The solid area fraction was determined from SEM images of the surface structures of the black scales of the snake. Single scales were fixed with double-sided carbon conductive tape (Plano, Wetzlar, Germany) on aluminum stubs. After sputter coating in a BAL-TEC SCD 500 Sputter Coater using a BAL-TEC QSG 100 Quartz Film Thickness Monitor (Bal-tec AG, Balzers, Lichtenstein) with 15 nm thick gold-palladium, the samples were studied in a scanning electron microscope Hitachi S-4800 (Hitachi High-Technologies Corp., Tokyo, Japan) at an accelerating voltage of 3 kV.

The solid area fraction of hierarchical structures, was measured at the micro- and nano-scale by using the software Image J 1.47. At the micro-scale the solid area fraction was determined from SEM images of four black scales. The apical surface of 106 leaf-like microstructures in relation to the whole area was determined. The solid area fraction at the nano-scale was calculated as the relation between the width of nanoridges and distances between them. Dimensions from 43 apical nanoridges were measured from eight different leaf-like structures of two scales.

### Calculation of the surface free energy of the scales

While the surface tension of liquids can directly be measured by tensiometry, the surface energy of solids has to be calculated indirectly from measurements of contact angles of liquid droplets on the solid in a vapour atmosphere (in this case air). Younǵs equation describes the relation of the contact angle θ of the liquid, the surface energies of the liquid (γl) and the solid (γs), and the interfacial energy at the interface of liquid and solid (γsl) at the three-phase-point. At this point the state of matter of vapour (v), liquid (l), and solid phase (s) adjoin [12].

In case of high-energy surfaces, spreading pressure resulting from molecules from the vapour phase that are adsorbed at the surface of the solid should be considered. For low-energy surfaces we can neglect these pressures and can use the following equation [13]:
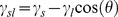
(1)


Before applying this equation for calculating the surface free energy (γs), residual variables are needed. The static contact angle (θ) was determined with a contact angle measurement. Using this data and the Owens–Wendt–Rabel–Kaelble-method (OWKR-method) [13]–[15] the surface free energy of the nearly unstructured ventral scales was calculated. Required surface tension data (SFT, γl) of polar and disperse components introduced by Fowkes [16] of all three fluids were taken from previous authors (water, diiodomethane [17], ethylene glycol [18]). All models and calculations that were applied in this study are described in detail in Z ˙enkiewicz [19].

### Contamination and fogging of scales

Self-cleaning properties of the scale surfaces were determined in accordance with the established method of Fürstner et al. [20]. Three pieces, each measuring ∼5×5 cm^2^ were cut out of the dorsal part of an exuvium of *B. rhinoceros* and were mounted on a flat plastic plate. With a digital camera (Olympus E-335, Olympus Deutschland GmbH, Hamburg, Germany) pictures were taken of the untreated exuvium-pieces. The plastic plates were placed horizontally on the ground of a box and were dusted with a fine layer of redwop-powder (Red-wop, Lightning Powder Company Inc, Jacksonville, USA). Redwop is a fluorescent, hydrophobic and highly adhesive powder. A UV light lamp (Omnilux black light bulb, energy saving, 25 W) was used to excite the fluorescence, and photographs were taken of the dusted specimens. These were then carefully transferred into a glass chamber that was connected to an ultrasonic fogger (Maxi fogger, Terra Exotica, Terra Exotica, Alfeld, Germany). The specimens were aligned in a 20° inclination with respect to the ground. The glass chamber with the specimens was fogged for 30 min. Specimens were then again photographed under the illumination of the black-light lamp. Black areas were measured with Image J 1.47 before and after the cleaning process.

Single scales of the contaminated and fogged specimens were cut out, mounted on aluminium stubs by using carbon conductive tape (Plano, Wetzlar, Germany) and covered with a 10 nm thick gold layer in a sputter coater (SCD-040, Balzers Union, Liechtenstein). Scanning electron micrographs (SEM) of the specimens were obtained by using an Oxford scanning electron microscope (Cambridge Stereoscan S200 microscope, Cambridge Instruments, Cambridge, UK).

## Results

### Contact angle measurements

**Figure 2:**
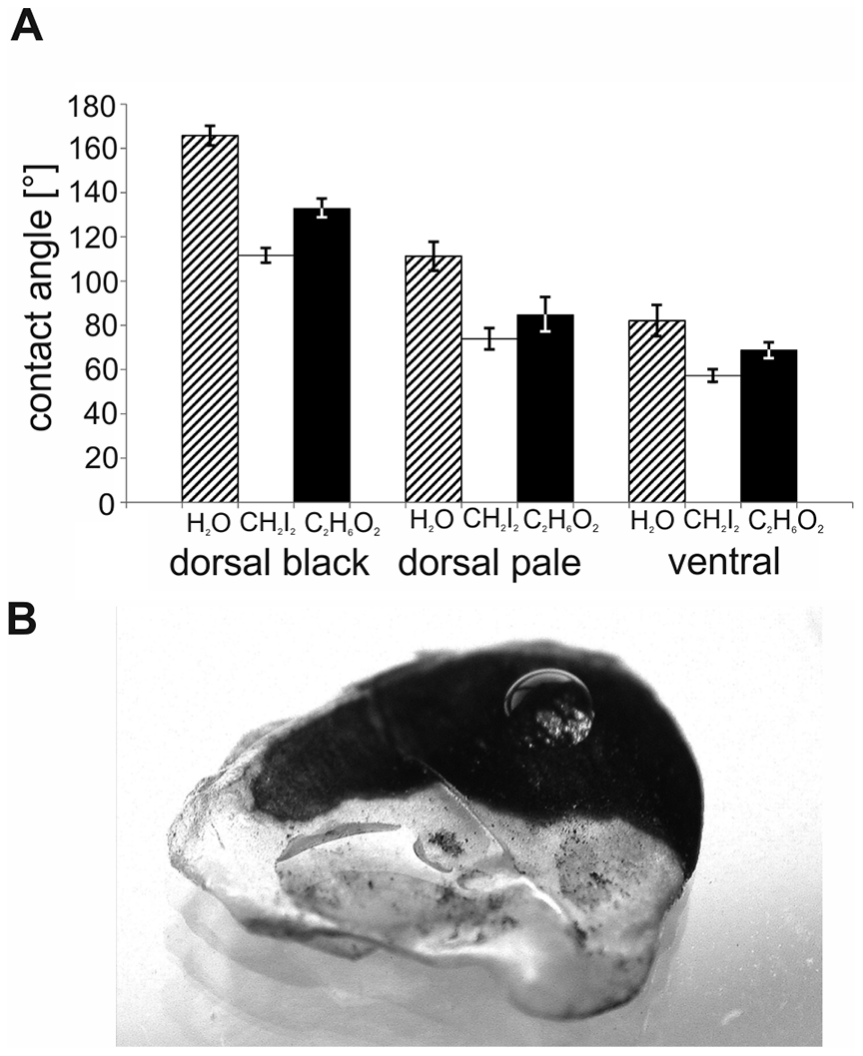
Contact angle measurements on scales of *B. rhinoceros*. **A,** Static contact angles of 1 µl droplets of water (H_2_O, hatched bars), diiodomethane (CH_2_I_2_, white bars), and ethylene glycol (C_2_H_6_O_2_, black bars) on ventral and dorsal pale and black scales on the exuvium of *B. rhinoceros*. Error bars indicate standard deviations of contact angles of ten individual measurements. **B,** Behavior of water droplets on the black- and white-coloured areas of the dorsal scale of *B. rhinoceros*. On the black area, the droplet remains in a spherical state due to the superhydrophobic properties of the underlying microstructure. In the pale area, the droplet spreads out and largely covers the area. The microstructure of this area has no superhydrophobic effect.

**Figure 3:**
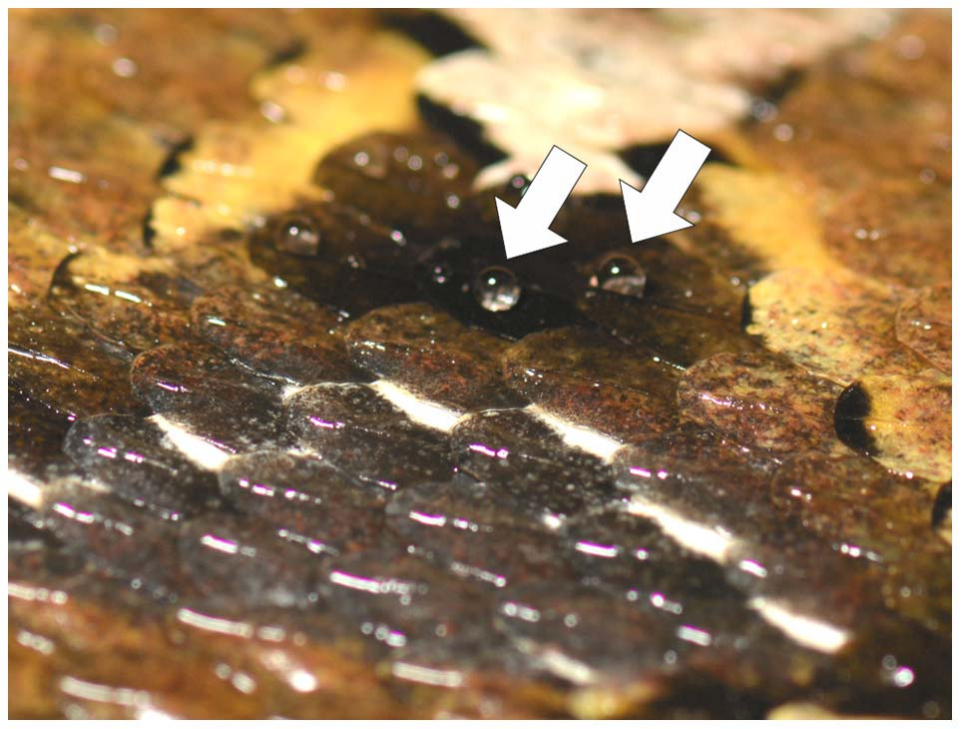
Photographic image of the skin of a living individual of *B. rhinoceros* after sprinkling with water. Whereas the rest of the skin is evenly wetted and shiny on black coloured scales appear black and matt further on, because they are not covered by water. There are only droplets in the keel regions of the scales (white arrows).

The different scale types of the West African Gaboon viper showed different wetting properties for the three liquids used (Fig. 2a, b). Our statistical tests (performed for each liquid separately) showed that static contact angles were significantly different on dorsal black, dorsal pale, and ventral scales (One-way ANOVA, P≤0.001, Holm-Sidak *post hoc*, P≤0.05). Black dorsal scales had significantly the highest static contact angles for all three liquids. Ventral scales had significantly the lowest static contact angles for all three liquids. Static contact angle of water was 166°±4.4° on ten different black dorsal scales, 111°±6.5° on ten pale dorsal scales, and 82°±7.0° on ten ventral scales (Fig. 2a, b). Similar results were observed in the live individual of *B. rhinoceros* (Fig. 3). Calculated surface free energy was 26.5 mN/m on ventral scales.

### Testing of the Cassie-Baxter wetting hypothesis

The validity of the Cassie-Baxter model for the snake skin material was tested in accordance to Bormashenko et al. 2007 [21]. Since surface of black scales is hierarchically structured, we used the following modification of the Cassie-Baxter equation [22] that considers superimposed surface structures of two different dimensions (see [21]):
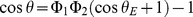
In this equation Φ_1_ is the ratio of apical surfaces of the leaf-like microstructures and Φ_2_ is the ratio of nanoridges to the whole area. From our measurements Φ_1_ was about 0.13 and Φ_2_ was about 0.12, thus the solid area fraction was about 0.016. With a contact angle in the range of that of nearly smooth ventral scales θ_E_ = 82° or the pale dorsal scales θ_E_ = 111°, the predicted apparent contact angle ranges from 169 to 172°.

### Contamination and fogging of scales

**Figure 4:**
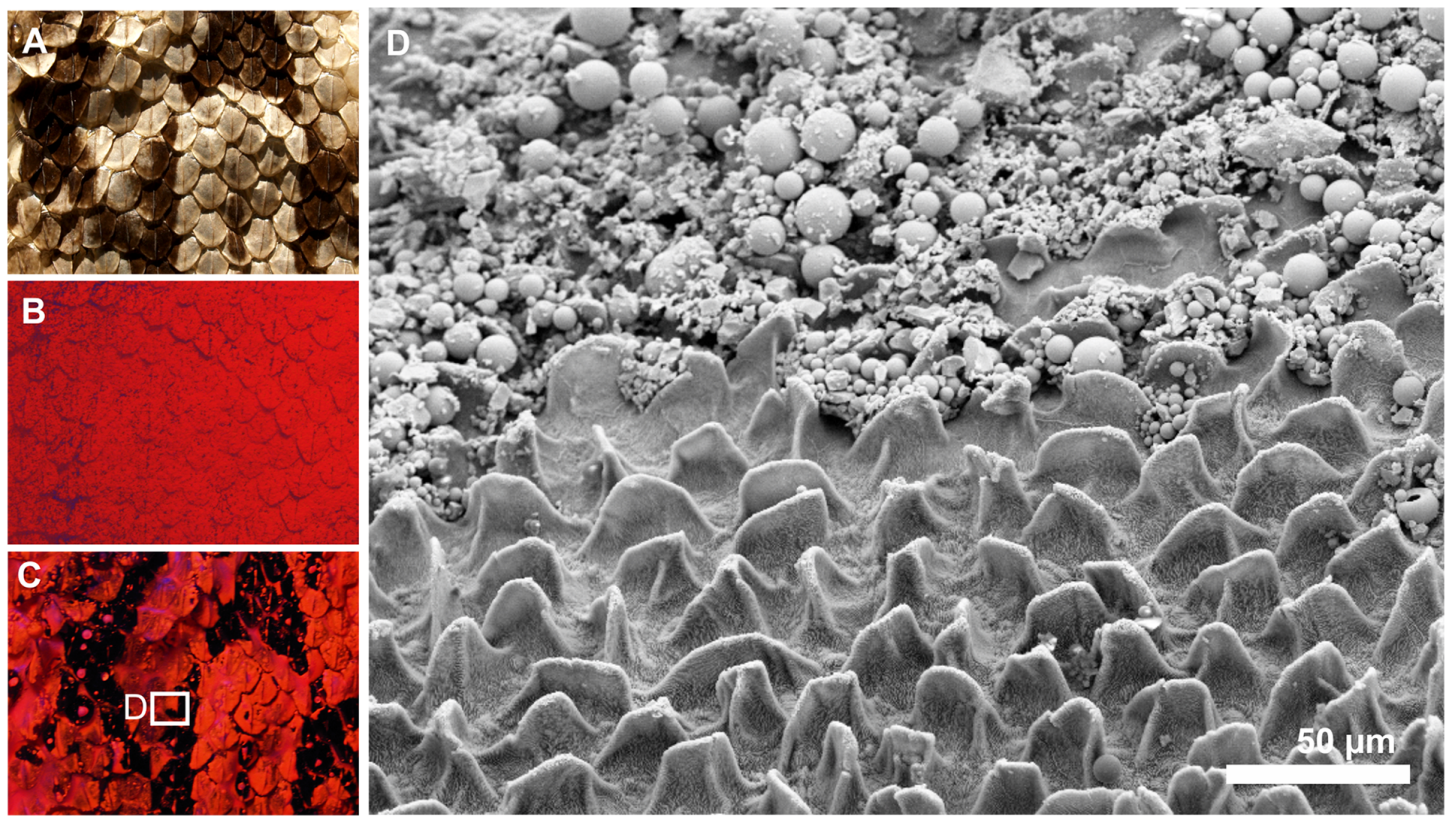
Self-cleaning ability of the skin of *B. rhinoceros*. **A–C,** Photographic images of an exuvium of *B. rhinoceros*. **A,** The exuvium at daylight before dusting it with the redwop. **B,** The exuvium under black light after dusting it with the redwop. **C,** The same exuvium under black light after fogging it for 30 min at an inclination of 20°. **D,** SEM-image of the *B. rhinoceros* scale with black and pale regions after contamination with the redwop dust and subsequent fogging. The (black) areas showing the characteristic micro- and nanostructure are free of the redwop particles. The (pale) areas showing the inconspicuous microstructure and no nanostructures are covered by a dense layer of the redwop particles.

After dusting the scales in the contamination chamber, black and pale coloured surface areas were evenly covered with a layer of the redwop dust (Fig. 4a, b). After fogging for 30 min, 89% of the black areas were free of the redwop. Residues of the redwop remained only in droplets that adhered to the keels of the scales (Fig. 4c). The pale areas remained completely covered with the redwop (Fig. 4c). SEM-images showed that the redwop dust formed a dense layer on the pale surface areas. The black surface areas were to a large extent free of the contaminating particles (Fig. 4d).

## Discussion

Before discussing the results and values obtained for the available exuvium, we would like to point out, that all exuviae used in this study showed traces of wear and usage. Presumably, we could have expected to obtain even higher contact angles for the skin of a freshly shed West African Gaboon Viper; in case there had not been any previous contact with the substrate and there would have been no contamination or friction marks.

The dorsal epidermis of the snake possesses black scale areas that show a complex micro- and nanostructure on their surface [1]. Little is known about the chemical composition of the skin of the West African Gaboon viper. Studies on boid, colubrid, and pythonid snakes revealed six layers of different types of keratin and cell shape in the epidermis [23], [24]. Although the thickness of these layers differs among species, the order of layers is the same in all species investigated [23]. Concluding from these studies, the outermost layer of ventral and dorsal scales of both colours, the oberhäutchen, consists of ß-keratin of 10–15 kDa [23], [24]. The only study addressing chemical composition of the West African Gaboon viper focuses on lipids [25]. In exuvia of *Bitis rhinoceros* ceramides, phosphatidylethanolamine, phosphatidylcholine, sphingomyelin, fatty acid, and cholesterol were detected [25]. However, a broad-based study on other snake species revealed, that these lipids are mainly present in the inner layers of the epidermis and not in the oberhäutchen [26].

Our CA measurements revealed that water droplets on the black areas had static contact angles higher than 160° and low roll-off angles. The contact angles on the adjacent pale scale areas were significantly lower (below 115°). Thus, the black scale areas might be considered as superhydrophobic [27]. Such a superhydrophobicity has been shown for the spiny scale surfaces of geckos [28], [29], but has never been demonstrated for the scales of snakes.

The intrinsic wettability of the bulk material is a common feature of all surfaces with enhanced hydrophobicity [30]. The intrinsic wettability of solid material can be measured on its smooth, unstructured surface. As none of the examined epidermal surfaces of the snake was completely smooth, the intrinsic wettability of the epidermal material can only be estimated. Measurements on ventral scales that are nearly unstructured and pale dorsal scales that were less rough than dorsal scales allowed us to estimate that the outermost epidermal scale material must have an intrinsic static contact angle for water of slightly above 80° and a surface free energy of about 26.5 mN/m. Thus, the Gaboon vipeŕs scale material itself tends to be only slightly hydrophobic. Our data correspond to values that were previously measured on the reverse side of exuviae of geckos [28], [29].

We can assume that material properties and chemical composition of the outermost surface layer of the pale scale areas and the black scale areas are very similar, as the structure and colour change from pale to black was observed even at the level of a single scale. We can therefore assume that the superhydrophobic properties of the black areas must result from their distinct micro- and nanostructure, which is less developed on the pale scale areas and absent on ventral scales.

The physical background of the role of surface structures in its wetting properties is well known [22], [30], [31]. Wenzel [31] showed that surface roughness can amplify the wettability of a surface in case of homogeneous wetting, i.e. when a liquid is wetting also the cavities of the surface roughness. The effect of roughness on the contact angle in case of inhomogeneous wetting, where air is entrapped in cavities between the solid and liquid (composite states), was described by Cassie and Baxter [21].

Several studies established theoretical criteria for geometries generating superhydrophobicity [2], [3], [5], [6], [8]–[10]. The theoretical criteria have also been proven experimentally [32]. Transfer of these criteria to biological surfaces is not trivial, as the majority of models and experimental data use simplified models of surface roughness. In these models, simple geometrical forms as pillars are arranged in a completely homogeneously array. In contrast, the superhydrophobic surface structure of the Gaboon viper is characterized by a high complexity of (rounded) shapes and their inhomogeneous, scattered array. However, the hierarchical arrangement of micro- and nanostructures of snake scales was assumed as advantageous for superhydrophobic surfaces in several studies [5], [10].

The dimensions of nanoridges fulfil the criteria for superhydrophobicity which were established in models that analyzed the thermodynamics of composite states of vapour and liquid phases: Extrand [2]–[3] used a model of rectangular shaped pillars. Based on this model, he introduced the `contact line density criterioń providing information about the contact area interactions between liquid and asperities and `pillar height criterioń (minimal pillar height) as criteria for superhydrophobicity. Li and Amirfazli [10] emphasized that a high aspect ratio (height/pillar distance) and small relative pillar width (width/pillar distance) are crucial criteria for high contact angles. The Gaboon vipeŕs leaf like structures are covered with 600 nm height and 60 nm thick nanoridges arranged in parallel to each other with a distance of 330 nm. The large relative height (height/distance of ridges  = 1.8), small relative width (thickness/distance of ridges  = 0.18) and high ratio of length and thickness (10) of the nanostructures comply with the models of Extrand [2], [3] and Li and Amirfazli [10].

This result is also confirmed by our calculations using the Cassie-Baxter equation [22] modified for hierarchical surface geometry [21]. The calculated apparent contact angle of 169–172° was only slightly higher than our experimentally determined contact angle obtained on dorsal black scales (166°±4.4°). The accordance of the model predictions and the experimental data provides strong evidence for a Cassie-Baxter wetting state in black scales of the West African Gaboon viper. The slight discrepancy between predicted and experimental data can be explained by the complexity of the biological samples that lack smooth, homogeneous surfaces and have traces of wear which makes it difficult to assess absolute values. On the fresh skin even higher experimental values in the range of the model can be expected. Furthermore, it should be also considered that the Cassie-Baxter equation is based on the intrinsic contact angle of the scale material (θ_E_). Since none of the scales of the snake has an ideal smooth surface, this variable could only be estimated from contact angle measurements on the less structured ventral and pale dorsal scales. Furthermore, due to the round shape of the black scaleś microornamentation, the ratios of apical surfaces of leaf-like structures and nanoridges to the whole area (Φ_1_ and Φ_2_) are only approximated values.

Our contamination and fogging experiment showed that water droplets on the black scale areas rolled-off at inclination angles of 20°, and droplets were able to carry away the hydrophobic redwop particles from the black scale areas. Thus, the hydrophobic particles adhered to the water droplets rather than to the epidermal surface. In contrast, the pale scale areas remained contaminated with the redwop particles. The alignment of the nanoridges suggests that droplets will roll-off in all directions (isotropic roll-off characteristics): the nanoridges on the leaf-like microstructures are orientated in all directions. The black scale areas also comply with theoretical considerations made by Marmur [7], who states that a combination of low roll-off angles and superhydrophobicity leads to self-cleaning properties.

Most artificial and biological superhydrophobic surfaces consist of pillars, rods, hairy structures or other tiny protuberances [33]–[38]. Also, the water-repellent scale regions of geckos are covered by spiny microstructures [28], [29]. In contrast, the black scale areas of the Gaboon viper possess small ridges that are arranged in parallel. While small sized parallel ridges are already known from the petals of some plants (cuticular folds [20]) or on butterfly wings [34], [36], [39], the ridges of the snake differ in some geometric traits from these models. The ridges of the snake have higher aspect ratio, interridge distances are smaller, and their alignment on microscopic leaf-like structures is multidirectional. The multidirectional alignment may lead to isotropic roll-off behaviour of water droplets, while the uni-directional alignment of the ridges on butterfly wings causes the anisotropic roll-off of droplets [39].

The West African Gaboon viper is a dweller of the African rainforest and bush lands [40]. In a recent study we showed that the alternating pale and black scale areas provide the snake with a superior camouflage [1]. We furthermore showed that the camouflage effect is enhanced by the micro- and nanostructuring of the black scale areas. Whereas the specific colour of ventral scales suggests, that their surface properties of these scales are more adapted to locomotion than to particular optical effects, the dorsal scales with their water repellency and self-cleaning properties can strongly contribute to the camouflage function. Under dry and clean conditions pigments and surface structures on black and pale skin surfaces generate a pattern of alternating areas with high and low reflectivity that is presumably adapted to diverse textures of the plant and ground material on the forest floor (disruptive colouration). Evenly distributed water droplets on the back sites of the skin would reduce the disruptive effect of this coloration pattern, because this would lead to a uniform appearance of the back. With its alternating wetting properties, the snake has an additional camouflage effect on the background of surrounding structures with different surface wettability. Additionally, water repellent surface structures on the black scales ensure that the pattern of regions with different reflectivity is also maintained under wet conditions. Black scales have a surface geometry that prevents water penetration of the interstices between the micro- and nanostructures which would impair the antireflective function. Specific optical properties for dry and wet conditions have been reported so far for arboreal species of bugs (Aradidae and Pentatomidae) [41]. The wetting properties of the integument of these insects are an adaptation to surrounding surfaces and aid in camouflaging these animals under changing wetting conditions of vegetation. In *B. rhinoceros*, also different cleaning properties of black and pale scales presumably assist the sustainable maintenance of contrast and result in strong optical adaptation to the habitat.

The pillar geometries already reported superhydrophobic self-cleaning structures in engineering and biology are vulnerable to mechanical damage. Our measurements were performed on shed skin that had been worn by the snake for more than two month. Although the tiny hierarchical structures were permanently exposed to mechanical stress, the snake skin still showed excellent water repellency and self-cleaning capability. The geometry of the surface structures could contribute to this robustness. The snakés nanoridges have a higher compactness than pillar structures, which lets expect a higher resistance against pressure and abrasion. Although the high aspect ratio of the nanoridges would be a great challenge to transform into an industrial design, the geometry could be an inspiration for technical surfaces. It is especially promising for multifunctional surfaces which must combine low-reflectivity, high absorbance, water-repellency, and self-cleaning properties. We are rather confident that viper surfaces described above could serve as a model for technological applications, such as solar heating of water, camouflage textiles, and optical instruments.
